# Conceptions of decision-making capacity in psychiatry: interviews with Swedish psychiatrists

**DOI:** 10.1186/s12910-015-0026-8

**Published:** 2015-05-21

**Authors:** Manne Sjöstrand, Petter Karlsson, Lars Sandman, Gert Helgesson, Stefan Eriksson, Niklas Juth

**Affiliations:** Center for Bioethics, Harvard Medical School, Boston, MA USA; Stockholm Centre for Healthcare Ethics, Department of Learning, Informatics, Management and Ethics, Karolinska Institutet, Stockholm, Sweden; Academy for Care, Work Life and Welfare, University College of Borås, Borås, Sweden and National Centre for Priority Setting in Health Care, Linköping University, Linköping, Sweden; Centre for Research Ethics and Bioethics, Department of Public Health and Caring Sciences, Uppsala University, Uppsala, Sweden

**Keywords:** Psychiatry, Bioethics, Mental capacity, Personal autonomy, Paternalism, Involuntary commitment

## Abstract

**Background:**

Decision-making capacity is a key concept in contemporary healthcare ethics. Previous research has mainly focused on philosophical, conceptual issues or on evaluation of different tools for assessing patients’ capacity. The aim of the present study is to investigate how the concept and its normative role are understood in Swedish psychiatric care. Of special interest for present purposes are the relationships between decisional capacity and psychiatric disorders and between health law and practical ethics.

**Methods:**

Eight in-depth interviews were conducted with Swedish psychiatrists. The interviews were analysed according to descriptive qualitative content analysis in which categories and sub-categories were distilled from the material.

**Results:**

Decision-making capacity was seen as dependent on understanding, insight, evaluation, reasoning, and abilities related to making and communicating a choice. However, also the actual content of the decision was held as relevant. There was an ambivalence regarding the relationship between psychiatric disorders and capacity and a tendency to regard psychiatric patients who made unwise treatment decisions as decisionally incapable. However, in cases relating to patients with somatic illnesses, the assumption was rather that patients who made unwise decisions were imprudent but yet decisionally capable.

**Conclusions:**

The respondents’ conceptions of decision-making capacity were mainly in line with standard theories. However, the idea that capacity also includes aspects relating to the content of the decision clearly deviates from the standard view. The tendency to regard imprudent choices by psychiatric patients as betokening lack of decision-making capacity differs from the view taken of such choices in somatic care. This difference merits further investigations.

**Electronic supplementary material:**

The online version of this article (doi:10.1186/s12910-015-0026-8) contains supplementary material, which is available to authorized users.

## Background

How to deal with patients who make seemingly unwise or irrational decisions is a central ethical problem in healthcare. According to the standard view of the normative role of autonomy in healthcare, respect for autonomy means that patients have a right to choose and a right to accept or decline information [1]. Hence, it is typically argued that autonomous decisions are to be respected and that patients have a right to reject proposed treatments, even if this may have negative consequences for the patient him or herself [1-3]. However, it is also argued that decisions that are insufficiently autonomous may justifiably be overruled if this is in the patient’s presumed best interest. Thus, it is a central issue for practical healthcare ethics to distinguish between decisions that are sufficiently autonomous to warrant respect, and decisions that may justifiably be overruled and other mechanisms for decision-making need to be sought [1-3].

In somatic healthcare, the concept of decision-making capacity is typically used to determine whether or not a particular healthcare decision should be respected [3-5]. Decision-making capacity (or decision-making competence), as it has been conceptualised in law and bioethics, is a compound of abilities typically divided into four sub-capacities: understanding, appreciation, reasoning, and choice [4,5]. This means, roughly, that a patient who is able to understand and evaluate information about his or her illness and the likely consequences of accepting or rejecting a proposed treatment is also seen as capable of making a decision about that treatment. The concept, however, is not undisputed, and it has been argued that capacity also includes a fifth category, i.e. having rational or autonomous (sometimes referred to as authentic) values and wants [4].

There is an extensive literature on philosophical aspects of decision-making capacity [4], and several empirical studies on standards of capacity and clinical assessments of capacity have been conducted [6-13]. However, there are, to our awareness, no previous studies on how clinicians conceptualise decision-making capacity and its relationship to psychiatric disorders, which is the focus of the present article.

### Decision-making capacity in psychiatry

Psychiatric disorders, particularly episodes of mania and psychosis, affect abilities typically associated with decision-making capacity [11,12]. However, even in severe cases of mental illness, decisional capacity may be retained [7,11-13]. In fact, the rate of decisional incapacity may be not be very different among psychiatric inpatients compared to general hospital inpatients [7,11].

In laws and regulations on psychiatric care, decision-making capacity typically does not have the same standing as in somatic care. Laws on involuntary psychiatric treatment typically focus on presence of (severe) mental illness, need of treatment, or danger to self or others – not on the patient’s ability to make autonomous decisions [2,14-16]. In contrast to this, it has been argued that lack of decision-making capacity should be a necessary criterion for involuntary treatment in psychiatric care just as in somatic care [2,16,17]. However, this idea is controversial, as it has been argued that patients with seemingly pathological or self-destructive wants may pass as competent according to standard criteria for capacity, even in cases when they pose a danger to themselves [4,18,19].

### The legal situation in Sweden

Patients in Sweden have a basic legal right to reject care and treatment. Medical care and treatment is, as a general rule, voluntary. However, this can under certain conditions be restricted. Most importantly, Swedish law makes exceptions in cases of serious psychiatric disorder and in cases of certain communicable infectious diseases [20,21]. Under the law on involuntary psychiatric treatment (*Lagen om psykiatrisk tvångsvård, LPT*), involuntary treatment can be given to patients if the following three criteria are met: (1) the patient has a serious psychiatric disorder, (2) the patient has an imperative need of psychiatric care, and (3) the patient refuses such care or is deemed unable to make a decision regarding the care [21]. The concept of serious psychiatric disorder primarily refers to psychotic disorders and depressions with a risk of suicide. However, what is important in practice is symptoms – not specific diagnoses. Hence, also psychiatric symptoms caused by a somatic condition (e.g. a neurological condition entailing psychotic symptoms) may qualify as a serious psychiatric disorder according to the law. The criterion ‘imperative need of psychiatric care’ includes situations where there is an imminent risk of harm to self or others, as well as situations where hospital admission is deemed necessary in order to ensure treatment of a serious psychiatric condition (for instance a patient with schizophrenia whose mental or physical condition is deteriorating). Also social circumstances and somatic illnesses may be taken into account when assessing a patient’s treatment need. The first and second criteria are in many cases intertwined. Hence, if a patient suffers from a psychiatric disorder and is deemed to have an imperative need of psychiatric inpatient care, the psychiatric disorder would typically also qualify as serious. Although decision-making capacity is mentioned in the law, lack of capacity is not a necessary requirement for involuntary treatment as a refusal to accept treatment is sufficient if the two first criteria are met [21,22].

The concept of decision-making capacity is not defined in Swedish healthcare legislation. In regulations issued by the National Board of Health and Welfare, decision-making capacity is mentioned in the context of end-of-life care, where it is stated that a patient who is decisionally capable has a right to reject life-supporting treatment [23]. The regulations state that, to assess capacity, the caregiver is obliged to make sure that the patient understands relevant information, is able to appreciate and understand the consequences of not initiating or of discontinuing the treatment, has had enough time for deliberation, and adheres to the decision after deliberation [23]. It is not clear, however, to what extent patients judged to have less than adequate decision-making capacity can be treated against their expressed wishes in such cases. Patients with psychiatric disorders *and* acute life-threatening somatic conditions may, however, be subjected to involuntary treatment of their somatic disorder if the general conditions for involuntary psychiatric treatment are also satisfied [22].

### Aims and rationale of the study

The aim of the present study is to explore psychiatrists’ conceptions of decision-making capacity and its practical role in Swedish healthcare, particularly focusing on psychiatric care. This is interesting for several reasons. Firstly, psychiatry is the only medical field where involuntary treatment for the sake of the patient is clearly sanctioned by Swedish law, while, in contrast to much discussion in contemporary bioethics, lack of decision-making capacity is not a necessary criterion for such treatment. Secondly, many psychiatric disorders affect abilities related to decision making, which means that psychiatrists often meet patients with impaired decision-making capacity. Thirdly, since the presence of a serious psychiatric disorder is necessary for compulsory treatment in Sweden, psychiatrists are often called in as consultants when patients make seemingly unwise healthcare refusals, also in somatic care. The uncertain legal and practical general role of decision-making capacity in Swedish healthcare makes it particularly interesting to investigate how Swedish psychiatrists’ reason regarding these issues.

## Methods

We conducted in-depth interviews with eight psychiatrists. The respondents were recruited continuously during the project, partly through chain-referral, according to a purposive sampling method [24], with the aim of covering views and experiences from psychiatrists with different backgrounds and experiences. Since this was an exploratory study in a largely unexplored field and since the interviews were rich in data, we decided that eight interviews would fulfil our aim at this point.

All respondents were working in the Greater Stockholm area at the time of the interview but several of them had previously worked in other parts of Sweden. Five were specialists in general psychiatry, two were also specialised in forensic psychiatry, and two had extensive experience in addiction psychiatry. Three of the respondents were residents in general psychiatry, in the middle or at the end of their residencies. Four were female and four were male.

All respondents were provided with information about the study, including information about the design and purpose of the study, that participation was voluntary, and that consent could be withdrawn anytime during the course of the study. The information was given in writing before the interview and repeated verbally at the beginning of the interview. The study was approved by the Regional Ethical Review Board in Stockholm (Dnr 2011/114-31).

### Interviews

The interviews were conducted in Swedish and started off from a semi-structured guide with nine main questions with the possibility of qualifying follow-up questions, depending on the course of the interview (Additional file 1). All participants were interviewed once. The interviews lasted about 1–1,5 hours and were recorded with a digital voice recorder and transcribed verbatim. The participants were asked how they perceived the concept of decision-making capacity and to describe cases where patients’ decision-making capacity had been called into doubt. The questions addressed their understanding of the concept, its relevance and relation to psychiatric disorders, and experiences of clinical cases relating to the issues. Questions relating to decisions about involuntary treatment were also addressed and are presented in a separate paper [25]. There were a large number of follow-up questions, and topics were also covered that are not listed in the interview guide. The participants were encouraged to use anonymised cases from their own clinical experience to elaborate on the questions.

### Qualitative analysis

The eight interviews were analysed using a qualitative descriptive content analysis as described by Sandelowski [26] to extract categories and subcategories from the content in the interviews. Initially the text was read repeatedly to get an overall impression of the content. Next, meaning units, phrases expressing thoughts relating to the overall research questions, were identified in the text. Meaning units expressing the same idea were then sorted together in sub-categories. The sub-categories were then sorted into wider categories [27,28]. The initial analysis was done inductively, without pre-determined categories, but the bioethical literature formed a theoretical framework that guided interpretation of the material and the formation of the main categories. Hence, when subcategories were in line with the main aspects of capacity described in the theoretical literature, the established concepts of understanding, appreciation, reasoning and choice were used [4,5].

## Results

The results cover the interviewees’ understanding of the concept of decision-making capacity and the factors they mentioned as important for the assessment of patients’ decision-making capacity. The categories and sub-categories identified are described below and instanced with quotations from the interviews (Table 1).

**Table 1:** **Categories and subcategories**

**Categories**	**Sub-categories**
Ability to understand information	
Appreciation of one’s situation	Insight into one’s condition
	Ability to recognise and evaluate the consequences of one’s decisions
Ability to reason one’s way to a decision	Ability to process thoughts logically
	Ability to integrate different viewpoints and arguments
Ability to make and communicate a stable choice	Ability to settle for a decision
	Ability to communicate the decision
	Ability to control impulses
Making a medically sound decision	
Decisional capacity and the law on involuntary treatment	Need for involuntary treatment as indicative of incapacity
	Having capacity despite having a serious psychiatric disorder

### Ability to understand information

An idea put forth by almost all respondents is that ability to understand information is necessary for decision-making capacity. This included general cognitive abilities to understand information about the condition, treatment, and outcomes.

*…that you’re able to understand what a physician says. That you suffer from renal failure or you suffer from psychosis. […]. But also that, er…you’re able to understand information about a treatment and what it means.*

-Resident in psychiatry, male.

One respondent told about a patient who had had a traumatic brain injury with severe cognitive sequelae.

*And there… there is nothing. You cannot have any kind of outcome-focused dialogue. It’s just impossible.*

*-*Specialist in psychiatry, female.

Although commonly associated with psychiatric disorders, lack of ability to understand information was not in itself seen as indicative of disorder.

*There are situations when the patient does not seem to be able to take in what you say. It could be that they are upset, it could be a crisis, or that they don’t have the intellectual resources. This does not imply that it should be labelled as a disorder*.

-Specialist in psychiatry, male.

### Appreciation of one’s situation

Insight into one’s illness and the ability to realise and evaluative the consequences of different alternatives were typically brought up as key elements of capacity.

#### Insight into one’s condition

Insight was mentioned by several interviewees, and described in terms of understanding the nature and gravity of one’s own illness and how it affects one.

*…she, she wasn’t… if you think about decision-making capacity then she wasn’t… she had no insight regarding what kind of illness she had.*

-Resident in psychiatry, male.

A distinction was sometimes made between patients in somatic care who show a lack of insight, and patients with psychiatric disorders and limited insight.

*Then there are many patients who do not understand the gravity of their illnesses, who are trivialising their illness. And you can do that, but the problem arises if you… at the same time, have a serious psychiatric disorder.*

-Specialist in psychiatry, male.

#### Ability to recognise and evaluate the consequences of one’s decisions

Several respondents stressed the importance of being able to foresee and evaluate the consequences of different actions and decisions, including seeing whether or not an outcome would be beneficial or harmful.

*I would rather say that it is about understanding the consequences of one’s decision. This decision that I make, what are the consequences of it?[…] Because it all boils down to:‘What’s it gonna be like for me’? That is also a consequence*.

-Specialist in psychiatry, female.

It was argued that decisional capacity could be affected by psychiatric disorders that altered a person’s appreciation of him- or herself and distorted the person’s ability to evaluate alternatives and outcomes. One example mentioned was depressive disorders.

*Then the answers would reflect rather nihilistic and depreciative views of himself and would be about it not mattering whether he was alive or not, because nobody ever really wanted him, and so on.*

-Resident in psychiatry, male.

### Ability to reason one’s way to a decision

This category covers the ability to use and process information in order to reach a decision, including the ability to take different viewpoints and arguments into account in one’s decision-making.

#### Ability to process thoughts logically

In order to be decisionally capable, it was argued that persons need the ability to process thoughts in a logical manner. One respondent described a patient with schizophrenia and thought disturbances who was argued to lack capacity.

*And that also includes thought disturbances. And…how you sort of have…an ability to think, in general.*

-Resident in psychiatry, female.

#### Ability to integrate different viewpoints and arguments

The ability to integrate different arguments and points of views was also mentioned as a criterion for capacity. One respondent mentioned a case of a young woman who, after a long discussion, had rejected anti-depressive treatment, despite signs of serious illness. The respondent argued that although the patient did not fully appreciate the possible consequences of her decision, she was nevertheless competent.

*And I think she was able to integrate rational and emotional arguments and to listen to what others had to say*.

-Resident in psychiatry, male.

### Ability to make and communicate a stable choice

This category covers aspects about the choice itself, i.e. to be able to settle for a decision and communicate it.

#### Ability to settle for a decision

One basic ability mentioned was to be able to decide for one or the other line of choice. One respondent mentioned a case where severe ambivalence resulted in a complete inability to make even basic decisions.

*If you are sufficiently depressed you may be unable to decide if you should put on your left sock first or your right sock. So you just sit there… And that a patient in that condition should, for instance, choose between this or that medication… They can’t do it.*

-Specialist in psychiatry, female.

#### Ability to communicate the decision

A further criterion discussed was the ability to communicate the decision.

*Another interesting group is patients with aphasia… there it can be quite difficult to know.*

- Resident in psychiatry, female.

One example quoted from outside the psychiatric practice was patients with so-called ‘locked-in’ syndromes, where almost all ability to communicate with the outside world is lost due to physical impairment.

*Patients with a locked-in syndrome are a sad business. Then it’s hard to communicate. Is there autonomy there? I don’t know. It presupposes a communicative ability as well.*

-Specialist in psychiatry, male.

#### Ability to control impulses

In a couple of instances, respondents mentioned the ability to control impulses as an element of decisional capacity. Although it was often related to the ability to evaluative consequences, it was also mentioned as a distinct ability. One respondent told about a patient with substance dependence and severe social problems:

*And she does not have much ability, to control her impulses. She has a personality disorder as well…*

When asked whether the patient was able to make decisions about her treatment, the respondent replied:

*I don’t think she had very much decision-making capacity really. To see the consequences of: If I do this, this happens, if I do that, that happens. She is so governed by her impulses*.

-Specialist in psychiatry, female

Another respondent told about a case where a patient had refused treatment for her depression. The patient was competent, the respondent argued, in part because of her ability to control and evaluate impulses before acting.

*I think she had the ability to resist sudden impulses*, *or at least to evaluate them.*

-Resident in psychiatry, male

### Making a medically sound decision

Decision-making capacity was sometimes linked to the actual decisions patients made. When patients made decisions that were perceived as medically sound, such as accepting treatment, they were seen as decisionally capable.

*I think it is a sort of decisional capacity, to ask for medication despite having a serious psychiatric disorder and delusions.*

-Specialist in psychiatry, female.

Conversely, it was typically assumed that psychiatric patients who rejected treatment were not decisionally capable.

*…for instance [patients with] paranoid schizophrenia, who are seriously ill and have…hear voices and have hallucinations and delusions and maybe feel pursued […] Then it feels like there is a strong need for care. And those who reject care… then… I don’t think they are competent.*

-Specialist in psychiatry, male.

This reasoning was only brought up in relation to patients with psychiatric disorders. When asked about patients in general medicine who did not comply with treatment recommendations, one respondent answered:

*Then I think that they do understand that. But they do it anyway. But they understand what the consequences are and are aware of them.*

-Specialist in psychiatry, female.

### Decisional capacity and the law on involuntary treatment

Many of the respondents made the law on involuntary psychiatric treatment their implicit starting point when discussing the issue of capacity. In reasoning about cases, the question of patients’ need for involuntary psychiatric treatment was often a central issue.

#### Need for involuntary treatment as indicative of incapacity

The respondents’ conceptions of incapacity were closely entwined with the legal criteria for involuntary treatment. One of the respondents said:

*Well in psychiatry so… ethics and…thoughts about decisional capacity, it drowns so to say in that you think…you constantly think according to LPT, which is the law on involuntary treatment. In a way you think more like…’is LPT applicable here?’*

*-*Resident in psychiatry, male.

Often, lack of capacity seemed to be presumed in psychiatric patients when involuntary treatment was deemed necessary. One respondent phrased this in the following way, referring to the Swedish law.

*A decision capable person is either: A: Not mentally ill or B: lacks an indispensable need for care.*

-Specialist in psychiatry, male.

As noted, in somatic care, on the other hand, capacity was typically assumed. One of the respondents argued that this difference was explained by differences in the law.

*However, there is no ground for not attributing decisional capacity to a patient in somatic care. There are no laws that make involuntary treatment possible. We have to assume that [somatic] patients are decisionally capable.*

-Specialist in psychiatry, male.

#### Having capacity despite having a serious psychiatric disorder

When the respondents were asked directly, it was typically argued that also patients with serious psychiatric disorders could have decisional capacity.

*… that is, even if you suffer from a serious psychiatric disorder, this doesn’t in itself imply that you are [incompetent].*

-Specialist in psychiatry, male.

This line of reasoning was also taken to imply that incapacity was variable and rarely all-encompassing.

*I believe there are people who have serious psychiatric disorders, but who are able to make correct decisions within some spheres [of their life].*

- Specialist in psychiatry, female

## Discussion

Several interesting issues concerning both the descriptive and the normative issues relating to decision-making capacity emerge from the interviews. Regarding what decision-making capacity is, the respondents gave answers tallying with standard bioethical literature on the subject. The extent to which the physicians’ and theorists’ conceptions of decision-making capacity correspond to each other is interesting, given that none of the respondents seemed to be familiar with the theoretical literature. Hence, ability to understand information and insight into one’s illness, along with ability to reason and ability to communicate a choice were held as key criteria and often mentioned in relation to clinical cases. However, these similarities notwithstanding, some interesting differences were found which we will discuss in more detail in the following sections. One notable example is the view that the content of the decision (i.e. accepting or refusing treatment) was held as a relevant factor, particularly in psychiatric cases. Another interesting issue that merits further discussion is the role of evaluative abilities, and the ability to control one’s impulses to act on one’s reasoned decisions.

When studying the results, it is important to bear in mind that the reasoning presented by the physicians was typically related to specific cases, and rarely elaborated in terms of general criteria or in terms of necessary or sufficient conditions. This also means that the material sometimes is ambiguous, sometimes because of semantic issues but also because the respondents were ambivalent about the substantial ethical issues that were discussed. This points to two things. Firstly, the questions discussed are highly complex, which also is reflected in previous studies and the theoretical literature on the subject. Secondly, although all respondents offered reasoned views regarding the concept of decision-making capacity, none of them directly referred to an established definition of the concept.

### Appreciation, volition and authenticity

Insight regarding one’s illness and need for treatment was typically considered a necessary criterion for decision-making capacity. Thus, capacity was not only conceptualised as an *ability* to understand, but actually appreciating and *holding* certain beliefs (about one’s medical condition and need for treatment). One major factor relating to insight concerned evaluative abilities: the ability to see one’s own needs and how one could benefit from treatment. Having values or goals is thus necessary for being able to weigh the risks and benefits of various alternatives, which is in line with Buchanan and Brock’s notion that competence requires a conception of what is good [3]. However, any attempt to include a more precise definition of this is likely to be controversial. For instance, it has been argued that decisional capacity requires a set of values or goals that is authentic to the decision-maker, and that decisions should be in line with these to qualify as competent [18,29]. The idea is controversial as both the precise meaning and normative relevance of authenticity are contested [2,29,30]. Hence, it is an interesting question whether the material included references to such ideas.

Firstly, the idea that making a medically sound decision is a criterion for capacity is not easily squared with the idea of authenticity, since authenticity concerns whether or not a certain decision is authentic to the decision-maker and not necessarily whether the decision is conducive to the decision-maker’s health or wellbeing [29]. Secondly, the ability to control impulses was linked to the ability to evaluate alternatives and outcomes, but was also mentioned as a distinct ability. Impulse control is typically not discussed in the standard literature on decision-making capacity [4,5,31]. Rather, it has been analysed as part of a separate capacity for voluntary choice [4,32]. Arguably, impulse control is conceptually distinct from evaluation and appreciation, as it concerns the ability to effectuate one’s decisions (i.e. to act upon a decision that is the result of an adequate decision making process). However, no additional account of authenticity is necessary to analyse this ability [33]. Thirdly, and perhaps most interesting with regard to authenticity, some respondents argued that capacity could be impaired by inadequate evaluative abilities, such as nihilistic or pessimistic evaluations of outcomes in a depressive disorder. In some cases, evaluative abilities may be deficient because of distorted factual assumptions, e.g. incorrigible beliefs that no one cares about whether or not the person lives or dies (as in the case described by one of the respondents above), that nothing will be better, or unreasonable beliefs about personal guilt [34]. However, it has also been argued that such deficiencies may be purely evaluative and exist despite adequate understanding of relevant facts [30]. One possible example of this could be a desire to remain severely underweight in certain cases of anorexia nervosa [18]. In such cases, or so it has been argued, a theory of authenticity could function to explain why a certain evaluation of a situation would be detrimental to capacity [18,30]. One problem with this idea is that it has proved notoriously difficult to define authenticity in a way that is both normatively reasonable and useful in a clinical setting [30]. However, it is not clear that the cases described in this study really concern this kind of evaluative deficiency.

### A difference between somatic and psychiatric patients?

In the interviews, patients with psychiatric disorders who made imprudent decisions (such as refusing treatment) were typically seen as lacking in decisional capacity. As noted, theories of decision-making capacity typically focus on the process of making a decision, rather than the substantial content or the outcome of the decision [4]. In line with this, it is typically argued that clinicians should not conclude that patients lack decision-making capacity just because they make a decision against medical advice [4,5]. Here, it is noteworthy that respondents sometimes directly related capacity to making a medically sound decision, which is hard to square with the notion of respect for autonomy in healthcare. This is an interesting finding in itself, but it is also important to note that the connection was explicitly made only in relation to psychiatric patients. In somatic care, incapacity was not presumed in cases where patients made imprudent choices.

Two grounds for this distinction can be hypothesised. Firstly, in line with knowledge of how psychiatric disorders affect mental abilities, one may assume that psychiatric patients are more likely to have impaired decisional capacity than somatic patients. Respondents seemed to hold this view: psychiatric disorders were generally seen as something adversely affecting decision-making capacity. Thus, the assertion that patients with serious psychiatric disorders lack capacity may be a useful heuristic. However, in reply to a direct question, it was often stated that psychiatric disorders did not automatically render persons incompetent, even in serious cases – or at least not in all areas of life. Conversely, it was acknowledged that incapacity was not always the result of a psychiatric disorder. This reveals, we think, a split vision among some of the respondents: in abstract one acknowledges that being subject to serious psychiatric disorders does not automatically render a patient incapable of making specific decisions regarding their care, but faced with concrete cases, the default position is that such disorder implies incapacity. Secondly, respondents sometimes seemed to equate the issue of decisional capacity with the question of whether or not involuntary treatment was justified. Thus, the in clinical cases there was a tendency to conflate the issue of whether a patient has a moral or legal *right* to make decisions about their care with the issue of whether they have the *mental capacity* to do so. In some answers, this equation was openly acknowledged. For instance, one respondent argued that clinicians, as there is no legal ground for treating somatic patients involuntarily, just have to assume them to be capable.

It may be debated whether the tendency to label imprudent decisions in psychiatric patients as incompetent should be seen as unduly paternalistic, or whether it is an appropriate concern in the light of how mental illness affects decision-making abilities. However, even though it might be correct that fulfilling criteria for involuntary treatment or having a serious psychiatric disorder often corresponds to a lack of decision-making capacity, this is a contingent relation and not a conceptual one. Hence, the heuristic reasoning entails a risk that incapacity is unjustly presumed in patients with mental illness.

Our results may indeed lend support to the well-known allegation of a double standard when comparing psychiatric and somatic patients [2,35]. This exceptionalism regarding psychiatric patients is arguably reinforced by the legal framework. Thus, an interesting hypothesis generated from the material is that the special legal framework for psychiatric care explains why imprudent decisions may be regarded as invalid to a greater extent among psychiatric patients.

### Limitations

The number of respondents is relatively small and the topics discussed were complex; hence, it is reasonable to assume that issues have been left uncovered. However, the results cover most aspects of capacity that have been addressed in the theoretical literature. As noted, the respondents’ answers about cases were sometimes vague or ambiguous. In some instances this was likely caused by ambivalence regarding the substantial ethical issues, but sometimes this may be the result of an uncertainty about the basic concepts. For instance, questions about capacity assessments were sometimes interpreted as questions about involuntary treatment decisions. In such instances, there was a tension between creating a supportive atmosphere during the interview (i.e. letting the respondents share their thoughts without interruptions) and gaining precise answers. Moreover, the sensitive character of the issues discussed may cause respondents to take defensive postures and answers may be adjusted to accord with what the respondents perceive as socially desirable. It is also important to point out that the qualitative design does not allow conclusions about representativeness. Although it may be assumed that much of the reasoning here presented is common in Swedish psychiatry, it is not possible to say *how* common. In order to do that, quantitative studies are needed.

## Conclusions

This interview study explored Swedish psychiatrists’ conceptions of decision-making capacity in psychiatry. While several responses from the interviewed psychiatrists were well attuned with mainstream bioethical literature on the subject, some interesting differences were also identified. Most notably, decision-making capacity was not understood as merely involving understanding of the situation and the ability to identify rational means to existing ends, but also, by some, as concerning those very ends, in particular that of the patient’s own health. However, the presumption that medically imprudent decisions were indicative of incapacity was only brought up in relation to psychiatric patients. Here, respondents tended to equate the issue of whether the patient had decisional capacity with the question of whether involuntary treatment was warranted. Hence, patients who met criteria for involuntary treatment were also regarded as decisionally incapable, despite the fact that lack of capacity is not a necessary legal criterion for involuntary psychiatric treatment in Sweden.

## Additional file

Additional file 1:
**Interview guide (Translated from Swedish).**

## References

[1] Beauchamp TL, Childress JF (2009). Principles of biomedical ethics.

[2] Tännsjö T. Coercive care: the ethics of choice in health and medicine. London; New York: Routledge; 1999.

[3] Buchanan AE, Brock DW. Deciding for others: the ethics of surrogate decision making. Cambridge; New York: Cambridge University Press.

[4] Charland LC. Decision-making capacity. In: Zalta EN, editor. The Stanford Encyclopedia of Philosophy (Fall 2014 Edition). URL http://plato.stanford.edu/archives/fall2014/entries/decision-capacity/%3E

[5] Appelbaum PS, Grisso T (1988). Assessing patients’ capacities to consent to treatment. N Engl J Med.

[6] Seyfried L, Ryan KA, Kim SY (2013). Assessment of decision-making capacity: views and experiences of consultation psychiatrists. Psychosomatics.

[7] Owen GS, Szmukler G, Richardson G, David AS, Raymont V, Freyenhagen F (2013). Decision-making capacity for treatment in psychiatric and medical in-patients: cross-sectional, comparative study. Br J Psychiatry.

[8] Marson DC, Earnst KS, Jamil F, Bartolucci A, Harrell LE (2000). Consistency of physicians’ legal standard and personal judgments of competency in patients with Alzheimer’s disease. J Am Geriatr Soc.

[9] Kim SY, Caine ED, Swan JG, Appelbaum PS (2006). Do clinicians follow a risk-sensitive model of capacity-determination? An experimental video survey. Psychosomatics.

[10] Grisso T, Appelbaum PS, Hill-Fotouhi C (1997). The MacCAT-T: a clinical tool to assess patients’ capacities to make treatment decisions. Psychiatr Serv.

[11] Okai D, Owen G, McGuire H, Singh S, Churchill R, Hotopf M (2007). Mental capacity in psychiatric patients: systematic review. British J Psychiatry.

[12] Owen GS, Richardson G, David AS, Szmukler G, Hayward P, Hotopf M (2008). Mental capacity to make decisions on treatment in people admitted to psychiatric hospitals: cross sectional study. BMJ.

[13] Lapid MI, Rummans TA, Poole KL, Pankratz VS, Maurer MS, Rasmussen KG (2003). Decisional capacity of severely depressed patients requiring electroconvulsive therapy. J ECT.

[14] Zinkler M, Priebe S (2002). Detention of the mentally ill in Europe–a review. Acta Psychiatr Scand.

[15] Fistein EC, Holland AJ, Clare IC, Gunn MJ (2009). A comparison of mental health legislation from diverse Commonwealth jurisdictions. Int J Law Psychiatry.

[16] Dawson J, Szmukler G (2006). Fusion of mental health and incapacity legislation. British J Psychiatry.

[17] Doyal L, Sheather J (2005). Mental health legislation should respect decision making capacity. BMJ.

[18] Tan J, Stewart A, Fitzpatrick R (2006). Competence to make treatment decisions in anorexia nervosa: thinking processes and values. Philosophy Psychiatry Psychology.

[19] Tan JO, Hope T (2006). Mental health legislation and decision making capacity: capacity is more complex than it looks. BMJ.

[20] SFS 2004:168 Smittskyddslag. Stockholm: Ministry of Health and Social Affairs; 2004.

[21] SFS 1991: 1128 Lag om psykiatrisk tvångsvård. Stockholm: Ministry of Health and Social Affairs; 1991.

[22] SOSFS 2008:18 Socialstyrelsens föreskrifter och allmänna råd om psykiatrisk tvångsvård och rättspsykiatrisk vård. Stockholm: The National Board of Health and Welfare; 2008.

[23] SOSFS 2011:7 Socialstyrelsens föreskrifter och allmänna råd om livsuppehållande behandling. Stockholm: The National Board of Health and Welfare; 2011.

[24] Tansey O (2007). Process tracing and elite interviewing: a case for non-probability sampling. PS: Political Science Politics.

[25] Sjöstrand M, Sandman L, Karlsson P, Helgesson G, Eriksson S, Juth N. Ethical deliberations about involuntary treatment in psychiatry: interviews with Swedish psychiatrists. 2015; doi: 10.1186/s12910-015-0029-5. 10.1186/s12910-015-0026-8 PMC4447019 25990948

[26] Sandelowski M (2000). Whatever happened to qualitative description?. Res Nurs Health.

[27] Graneheim UH, Lundman B (2004). Qualitative content analysis in nursing research: concepts, procedures and measures to achieve trustworthiness. Nurse Educ Today.

[28] Malterud K (2001). Qualitative research: standards, challenges, and guidelines. Lancet.

[29] Juth N (2005). Genetic information values and rights: the morality of presymptomatic genetic testing.

[30] Sjöstrand M, Juth N (2014). Authenticity and psychiatric disorder: does autonomy of personal preferences matter?. Med Health Care Philos.

[31] Grisso T, Appelbaum PS (1998). Assessing competence to consent to treatment: a guide for physicians and other health professionals.

[32] Roberts LW (2002). Informed consent and the capacity for voluntarism. Am J Psychiatry.

[33] Juth N (2005). Genetic information values and rights: the morality of presymptomatic genetic testing.

[34] Hindmarch T, Hotopf M, Owen GS (2013). Depression and decision-making capacity for treatment or research: a systematic review. BMC Med Ethics.

[35] Hope RA (2004). Medical ethics: a very short introduction.

