# Supplementary material for: Conceptions of decision-making capacity in psychiatry: interviews with Swedish psychiatrists
Source: BMC Med Ethics. 2015 May 21;16:34. doi: 10.1186/s12910-015-0026-8 (PMC4447019; doi:10.1186/s12910-015-0026-8)
Supplement: Additional file 1: — Interview guide (Translated from Swedish). [file 12910_2015_26_MOESM1_ESM.docx]

**Interview guide**

(Translated from Swedish)

1) In your clinical work, do you encounter situations you consider ethically complicated?

- Can you give examples?

- How do you reason in these situations?

2) In your practice, do you encounter patients you consider unable to make decisions regarding their care?

- Examples: psychiatric care

- Examples: somatic care

3) What abilities do you consider important for being able to make decisions about care and treatment? Do they vary from one situation to another?

4) Is it possible to imagine a person who is incapable of making decisions regarding their care/treatment without suffering from any serious illness/disorder (psychiatric disorder or other, e.g. dementia)? Can you give examples?

5) Is it possible to imagine a patient suffering from a serious illness/disorder (psychiatric or other), but nevertheless being capable of making decisions concerning their own care?

6) Do you consider it sometimes justified to treat patients against their will? (alt. not act according to the patients decisions/wishes)

- If so, when? Does decision-making capacity matter?

- Do you think there is a difference between psychiatric and somatic illnesses/disorders in this respect?

7) To what extent do you consider patients’ self-determination to be respected in your field? Do you think it should be otherwise?

8) How would you describe your interest in ethics/moral philosophy?

9) Is there anything further you want to talk about?

-If the term autonomy is mentioned, ask how the respondent understands the concept.
